# Effects of short-term synaptic plasticity mechanisms on the dynamics of the network conductances

**DOI:** 10.1186/1471-2202-15-S1-P150

**Published:** 2014-07-21

**Authors:** Catalina Vich, Paolo Massobrio, Antoni Guillamon

**Affiliations:** 1 Department of Mathematics and Computer Science, Escola Politècnica Superior, Universitat de les Illes Balears, Mallorca, Palma, 07122, Spain; 2 Department of Informatics, Bioengineering, Robotics, System Engineering (DIBRIS), University of Genova, Genova, Italy; 3 Department of Applied Mathematics I, EPSEB, Universitat Politècnica de Catalunya, Barcelona, Spain

## 

In this work, we analyzed the effects that different levels of short-term synaptic facilitation and depression cause on the dynamics of the conductances in a network of excitatory and inhibitory neurons.

For this purpose, we added the short-term plasticity mechanisms for depression and facilitation [2] to the biophysical network model described in [1]. This model is made up of a population of excitatory and inhibitory multi-compartment neurons containing different membrane channels modeled according to the Hodgkin-Huxley formalism. Neurons have been spatially arranged on a line to emulate the connectivity rule experimentally observed in visual cortex. The synaptic transmission has been mediated by excitatory AMPA and NMDA, and inhibitory GABA currents.

Depending on the level of depression, the dynamics of the network presents two different behaviors: (i) a regime of up and down states; (ii) tonic activity [3]. By plotting the firing rate of excitatory versus inhibitory neurons, our results show that, without depression, the model seems to present a periodic orbit closed to one saddle node and one attractor. The possible saddle node, which corresponds to the (0,0) point is reached and maintained for a couple of seconds by the spontaneous dynamics causing down states. On the contrary, with respect to the possible attractor, by increasing the level of depression, the trajectory turns around it inducing oscillations in the firing rate scenario. For these levels, the trajectory reaches the saddle causing the tonic firing. We defined as *critical value* the minimum level of depression that switch the network behavior.

The critical value of the depression plays an important role in the changes of the conductances. As the depression level decreases we observe that the excitatory and inhibitory conductances exponentially increase (the goodness of fit presents an R^2^ coefficient greater than 0.8) for all levels of depression which are not close to the critical value.

On the other hand, the network shows a very different dynamics under the presence of short-term facilitation. In this case, we observed that for a low probability of release the network is active only for few seconds with a low firing rate. By increasing the level of facilitation of the network, we increased the probability to achieve up states with higher firing rate. These two different behaviors could be explained by observing that for low values of facilitation the AMPA conductances are almost zero. No claims can be inferred in the conductance values by changing the facilitation level since the data cannot be fitted by any kind of curve.

Finally, both facilitation and depression were treated together to see the joint effects on the network dynamics.
